# Ozone and possible heat effects on out-of-hospital cardiac arrest: a time-series study

**DOI:** 10.3389/fpubh.2026.1871041

**Published:** 2026-08-20

**Authors:** San-Gai Pan, Hai-Ping Zhang, Kai-tao Li, Shu-Hao Zhang, Run-kun Shen, Bin Gao, Ye Yuan, Wen-rui Zou

**Affiliations:** Tianjin Emergency Medical Center, Tianjin, China

**Keywords:** air pollution, distributed lag non-linear model, environmental epidemiology, out-of-hospital cardiac arrest, ozone, temperature, time-series analysis

## Abstract

**Background:**

Out-of-hospital cardiac arrest (OHCA) is a time-critical emergency with high mortality. Short-term environmental exposures, including temperature and air pollution, may trigger acute cardiovascular events, but their independent and lagged effects in northern Chinese cities remain unclear.

**Methods:**

We conducted a time-series study of 7,996 OHCA cases recorded by Tianjin's prehospital emergency system from July 2023 to June 2024. Daily OHCA counts were linked with meteorological and air pollution data. A distributed lag non-linear model with a quasi-Poisson link estimated non-linear and lagged effects of daily mean temperature (lag 0–14). Six pollutants (PM_2_.5, PM10, NO_2_, SO_2_, CO, O3) were examined in single- and two-pollutant models using lag0–1 exposures, adjusting for meteorological and temporal covariates.

**Results:**

Daily OHCA averaged 21.9 cases. Temperature showed a monotonic increase in cumulative risk, with a minimum risk temperature of −1.9 °C. High temperatures showed a possible short-term association„ peaking at lag 1 (RR ≈1.57 at the 95th percentile), whereas low temperatures showed no significant effect. Confidence intervals included unity, indicating limited statistical certainty. Ozone was the only pollutant consistently associated with OHCA. Each IQR increase in lag0–1 O3 corresponded to a 15.3% higher risk. In sex-stratified analyses, the effect was comparable in men (RR = 1.22, 95% CI: 1.08–1.39) and women (RR = 1.22, 95% CI: 1.04–1.43). Heat-related risks appeared stronger in women (RR = 3.16) than in men (RR = 1.58), though estimates were imprecise. Age-specific analyses were limited by sparse data, but in the ≥75-year group ozone showed a positive trend. Interaction analyses suggested ozone may amplify heat effects, with a significant interaction (*p* = 0.043). Lag-response analyses confirmed a short-term peak at lag 1 day in the high-ozone group.

**Conclusion:**

Elevated ozone concentrations are important short-term triggers of OHCA in Tianjin, while high ambient temperatures showed a possible short-term association with limited statistical certainty. Ozone effects were robust across sexes, while heat-related risks appeared stronger in women. Age-specific analyses were inclusinve, underscoring the need for larger cohorts. Exploratory analyses suggested a possible ozone–temperature interaction, but given imprecision and lack of multiple-testing adjustment, this finding should be interpreted cautiously.

## Introduction

Out-of-hospital cardiac arrest (OHCA) remains one of the most time-critical and lethal cardiovascular emergencies worldwide. Globally, the incidence of out-of-hospital cardiac arrest (OHCA) is estimated at approximately 30–60 cases per 100,000 population, with survival to hospital discharge generally below 10% (1, 2). Large registry studies in Europe and North America report survival rates of 6–12%, whereas in many Asian countries survival remains markedly lower, often around 2–4% (2, 3). In China, recent multicenter registry data indicate an OHCA incidence of about 37–40 per 100,000 population, with survival to hospital discharge close to 1% (4, 5). These figures underscore the substantial public health burden and the urgent need for improved prevention and emergency response strategies. Despite advances in prehospital systems of care, survival rates remain low, and the majority of events occur suddenly and without warning (1). Identifying modifiable short-term triggers of OHCA is therefore essential for improving early prevention, guiding public health alerts, and optimizing emergency medical services (EMS) preparedness (6). In recent years, environmental exposures—particularly ambient temperature and air pollution—have emerged as potential acute triggers of cardiovascular instability (7), yet their independent and combined effects on OHCA remain incompletely understood.

A growing body of epidemiologic evidence suggests that extreme temperatures can precipitate acute cardiovascular events through mechanisms such as thermoregulatory stress, dehydration, electrolyte imbalance, increased cardiac workload, and autonomic dysfunction (6, 8, 9). Both heat and cold have been implicated as potential triggers of myocardial ischemia, arrhythmias, and sudden cardiac arrest. However, the shape of the temperature–OHCA association varies across geographic regions, climate zones, and population characteristics. Moreover, temperature effects often exhibit delayed or lagged patterns, making distributed lag non-linear models (DLNM) an essential tool for capturing these complex exposure–response relationships (7, 10).

Air pollution has also been linked to acute cardiovascular morbidity and mortality. Fine particulate matter (PM2.5), nitrogen dioxide (NO_2_), carbon monoxide (CO), and ozone (O3) have been associated with arrhythmogenesis, endothelial dysfunction, systemic inflammation, and oxidative stress (11–14). Yet, evidence regarding their short-term effects on OHCA remains inconsistent. Some studies report positive associations for PM2.5 or NO_2_, while others find null or weak effects (6, 12, 13). Ozone, in particular, has shown heterogeneous associations across regions (6, 13), possibly due to its unique photochemical formation and inverse seasonal pattern relative to other pollutants. Furthermore, the interplay between temperature and air pollution complicates interpretation, and few studies have simultaneously evaluated both exposures using robust lag-structured models.

Northern Chinese cities such as Tianjin experience substantial seasonal variability, with cold, dry winters and hot, humid summers, alongside pronounced fluctuations in air pollution levels (15, 16). These environmental characteristics provide a unique natural setting to investigate short-term environmental triggers of OHCA. However, evidence from this region remains limited, and no prior study has comprehensively evaluated both temperature and multiple pollutants using DLNM within a full-year time-series framework.

To address these gaps, we conducted a population-based time-series study using 7,996 OHCA cases recorded by the municipal EMS system in Tianjin from July 2023 to June 2024. We applied a DLNM with quasi-Poisson regression to characterize the non-linear and lagged effects of temperature on OHCA risk, and we evaluated six major air pollutants using single-pollutant and two-pollutant models. We further performed extensive sensitivity analyses and seasonal stratification to assess the robustness of our findings. Our objective was to provide a comprehensive assessment of short-term environmental determinants of OHCA and to generate evidence that may inform public health preparedness and environmental risk mitigation strategies.

## Methods

### Study design and population

We conducted a population-based time-series study using all out-of-hospital cardiac arrest (OHCA) cases recorded by the Tianjin municipal prehospital emergency medical system between July 1, 2023 and June 30, 2024. During this 366-day period, 7,996 OHCA cases remained after data cleaning, which excluded non-medical causes of arrest, traumatic cases, duplicate records and records with incomplete date information. Each record contained demographic characteristics, date and time of symptom onset or emergency call, geographic location, prehospital treatment details, and resuscitation outcomes. Cardiac-origin OHCA was defined strictly as cases with prodromal chest pain, chest tightness, or palpitations prior to arrest. Other symptoms such as dizziness, syncope, transient loss of consciousness, or upper abdominal pain may also reflect cardiac causes (e.g., acute myocardial infarction, malignant arrhythmia), but EMS personnel cannot reliably classify these at the prehospital stage. This strict definition likely underestimates the true proportion of cardiac-origin OHCA and represents a methodological limitation. For the present analysis, OHCA cases were aggregated by date to generate daily OHCA counts.

### Ethical considerations

This study was approved by the Ethics Committee of Tianjin Emergency Medical Center (Approval No.TJEMC-2024-003). All patient data were anonymized prior to analysis. The study complies with the Declaration of Helsinki and local regulations governing retrospective research. Ethics approval was obtained, and patient consent requirements were waived due to the retrospective design.

### Environmental exposure assessment

Daily meteorological and air pollution data were obtained from five fixed-site monitoring stations located in the central urban district and four surrounding districts (east, west, south, and north). For each pollutant and meteorological variable, we calculated the citywide daily average across all five stations to represent population-level exposure. The following pollutants were included: PM2.5, PM10, NO_2_, SO_2_, CO, and O3. Ozone exposure was defined as the citywide daily 24-h mean concentration, averaged across five fixed-site monitoring stations. Meteorological variables included daily mean, minimum, and maximum temperature; diurnal temperature range; relative humidity; wind speed; and air pressure.

A standardized date variable was constructed from year, month, and day fields in the OHCA dataset. Daily OHCA counts were merged with environmental data by date to form a complete time-series dataset. A continuous sequence of dates covering the entire study period was generated to ensure no missing days; dates without OHCA events were assigned a value of zero. Missing meteorological values (1–2 days) were imputed using linear interpolation (na.approx function, zoo package). Additional derived variables included day of week (seven-level factor), public holiday indicator (binary), month, year, and a continuous time index for long-term trend adjustment.

### Descriptive analysis

We summarized daily OHCA counts, pollutant concentrations, and meteorological variables using means, standard deviations, medians, interquartile ranges (IQR), and minimum and maximum values. Correlations among pollutants were assessed using both Spearman rank correlation and Pearson correlation coefficients. Time-series plots with LOESS smoothing were generated to visualize seasonal patterns in OHCA incidence and environmental exposures.

### Distributed lag non-linear model for temperature

We applied a distributed lag non-linear model (DLNM) to characterize the non-linear and lagged association between daily mean temperature and OHCA risk. A cross-basis function was constructed to simultaneously model the exposure–response and lag–response dimensions. In the exposure dimension, natural cubic splines were used with internal knots placed at the 10th, 50th, and 90th percentiles of temperature (−1.92 °C, 17.02 °C, and 28.86 °C). In the lag dimension, natural cubic splines were applied with a maximum lag of 14 days, and internal knots were placed at log-spaced intervals (0.91, 2.27, and 5.64 days).

The minimum risk temperature (MRT) was identified by computing cumulative relative risks (RRs) across the full temperature range (0.1 °C increments) and selecting the temperature with the lowest cumulative RR. All temperature-related results were expressed as relative risks with 95% confidence intervals (CI).

### Air pollution models

To estimate the short-term effects of air pollution, we incorporated each pollutant into the DLNM model as a linear term. The primary exposure metric was the lag0–1 average (mean of same-day and previous-day concentrations), with lag0 (same day) exposures evaluated in secondary analyses. Effect estimates were expressed as the relative risk associated with each IQR increase in pollutant concentration. IQRs were calculated from the 25th to 75th percentiles of pollutant distributions during the study period.

### Two-pollutant models

To assess the independence of pollutant effects and evaluate potential confounding by co-pollutants, we constructed three two-pollutant models including combinations of PM2.5, NO_2_, and O3 (PM2.5 + O3; NO_2_ + O3; PM2.5 + NO_2_). Both pollutants were entered as lag0–1 linear terms. Model structure and covariate adjustments were identical to the single-pollutant models.

### Sensitivity analyses

A series of sensitivity analyses were conducted to evaluate the robustness of the primary findings and to assess the influence of key modeling assumptions. First, we examined the impact of alternative lag structures for temperature by refitting the DLNM using maximum lag periods of 7 and 21 days, in addition to the 14-day lag used in the main analysis. This allowed us to determine whether the estimated temperature effects were sensitive to the choice of lag window. Second, we varied the degrees of freedom used to control long-term and seasonal trends, testing models with 6, 7 (main model), 8, and 10 degrees of freedom per year. This analysis assessed whether different specifications of temporal adjustment materially altered the estimated associations. Third, we evaluated the influence of extreme weather conditions by excluding days with mean temperatures below the 1st percentile or above the 99th percentile and re-estimating the temperature–OHCA association. Fourth, we assessed the sensitivity of pollutant effects to alternative lag definitions by estimating associations using lag0, lag1, lag0–1, and lag0–3 exposure windows. Finally, to explore potential seasonal heterogeneity, we conducted stratified analyses for the cold season (November–March) and warm season (May–September), applying simplified DLNM specifications appropriate for the reduced sample size in each season. April and October were treated as transitional months with unstable climatic patterns and limited sample size; they were included in the overall analysis but not analyzed separately in seasonal stratification. Across all sensitivity analyses, model structures and covariate adjustments were kept consistent with the main analysis unless otherwise specified.

### Statistical analysis

All statistical analyses were performed using R version 4.5.1 (R Foundation for Statistical Computing, Vienna, Austria). Data preprocessing, and variable construction were conducted using the *dplyr* and *tidyr* packages, with date parsing handled by the *lubridate* package. Environmental data were imported via *readxl*, and outputs exported with *writexl*.

Distributed lag non-linear models (DLNM) were implemented using the *dlnm* package to construct cross-basis functions for temperature and generate exposure–lag–response surfaces.

We fitted quasi-Poisson generalized linear models with a log link to account for overdispersion in daily OHCA counts. The general model equation was: log[E(Yt)] = α + cb(Temp) + β1 · PM2.5t + β2 · PM10t + β3 · NO_2_t + β4 · SO_2_t + β5 · COt + β6 · O_3_t + ns(RHt, df = 3) + ns(APt, df = 3) + ns(WSt, df = 3) + ns(Timet, df = 7/year) + DOWt + Holidayt, where Yt is the daily OHCA count, cb(Temp) is the cross-basis function for temperature, RH is relative humidity, AP is air pressure, WS is wind speed, ns(Time) is the natural spline for long-term trend, DOW is day of week, and Holiday is a binary indicator.

For the temperature dimension, a natural spline was applied with knots at the 10th (−1.92 °C), 50th (17.02 °C), and 90th (28.86 °C) percentiles of the distribution. For the lag dimension, a natural spline with a maximum lag of 14 days was used, with log-equally spaced knots at 0.91, 2.27, and 5.64 days.

Covariates included relative humidity (natural spline, 3 df), air pressure (natural spline, 3 df), wind speed (natural spline, 3 df), long-term and seasonal trends (natural spline of time index, 7 df per year), day of week (six dummy variables), and public holidays (binary).

Model diagnostics included estimation of the overdispersion parameter (ϕ ≈1.118) and evaluation of residual autocorrelation using Durbin-Watson statistics and partial autocorrelation plots, which indicated no significant serial correlation.

For interaction analyses, ozone was stratified at the median daily concentration (89.2 μg/m^3^) into “low ozone” and “high ozone” strata, and modeled on the multiplicative relative risk scale. Visualization of exposure–response curves, lag effects, and DLNM surfaces was performed using *ggplot2* and built-in plotting functions within *dlnm*.

## Results

### Overview of study population and environmental conditions

**Figure 1:**
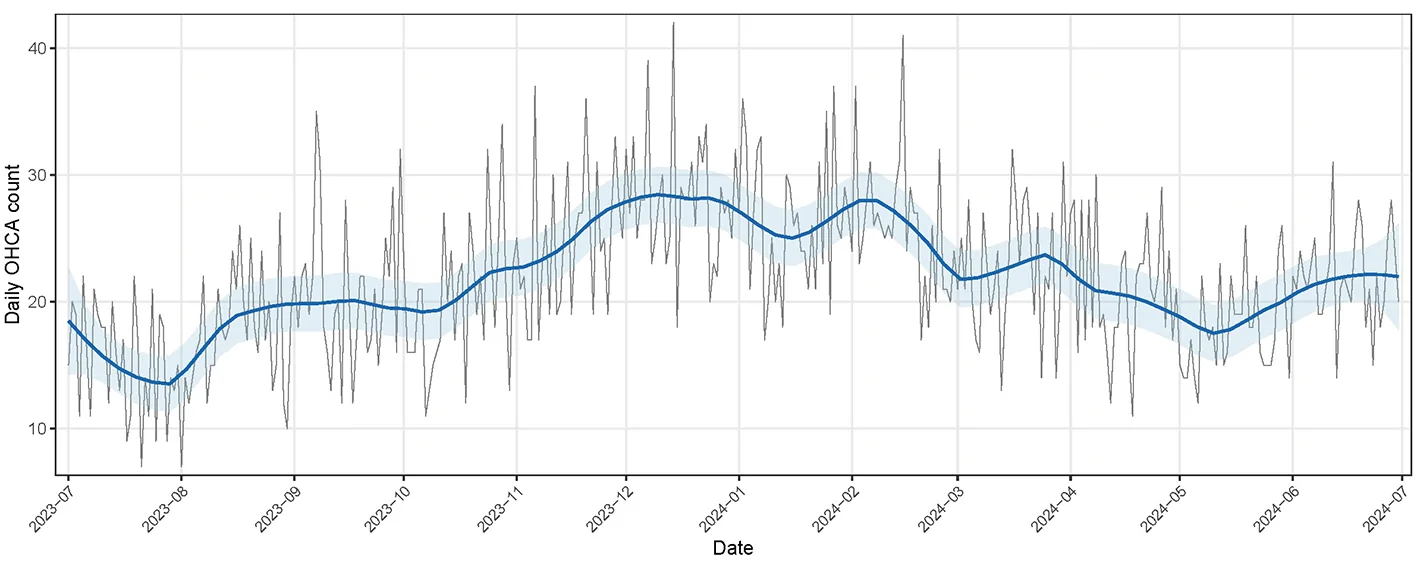
Daily out-of-hospital cardiac arrest (OHCA) counts with a locally estimated scatterplot smoothing (LOESS) trend line. Gray lines represent daily OHCA counts from July 1, 2023 to June 30, 2024. The blue LOESS-smoothed curve and its 95% confidence band illustrate the overall temporal trend and pronounced winter peak in OHCA incidence.

During the 366-day study period from July 1, 2023 to June 30, 2024, a total of 7,996 out-of-hospital cardiac arrest (OHCA) cases were recorded and aggregated into daily counts for time-series analysis. Daily OHCA incidence exhibited substantial temporal variability, with a mean of 21.85 cases per day (SD 6.13), a median of 21 cases, and a range of 7–42 cases. A pronounced seasonal pattern was observed, with markedly elevated OHCA counts during the winter months (December–February), where daily counts frequently exceeded 25–28 cases, contrasted with lower incidence during the summer months (July–September), typically ranging from 15 to 20 cases. This seasonal fluctuation was clearly reflected in the LOESS-smoothed trend (Figure 1). Demographic characteristics of OHCA patients showed that 65.2% were male, with a mean age of 71 ± 15 years. The majority of arrests occurred at home (86.8%) and within central urban districts (61.9%). Subgroup case numbers were: men (*n* = 5,211), women (*n* = 2,785); < 65 years (*n* = 2,198), 65–74 years (*n* = 2,308), ≥75 years (*n* = 3,490). A cardiac origin was identified in 1.8% of cases, and the first documented rhythm was ventricular fibrillation/ventricular tachycardia in 4.7%. Bystander cardiopulmonary resuscitation was performed in 19.4% of patients.

**Figure 2:**
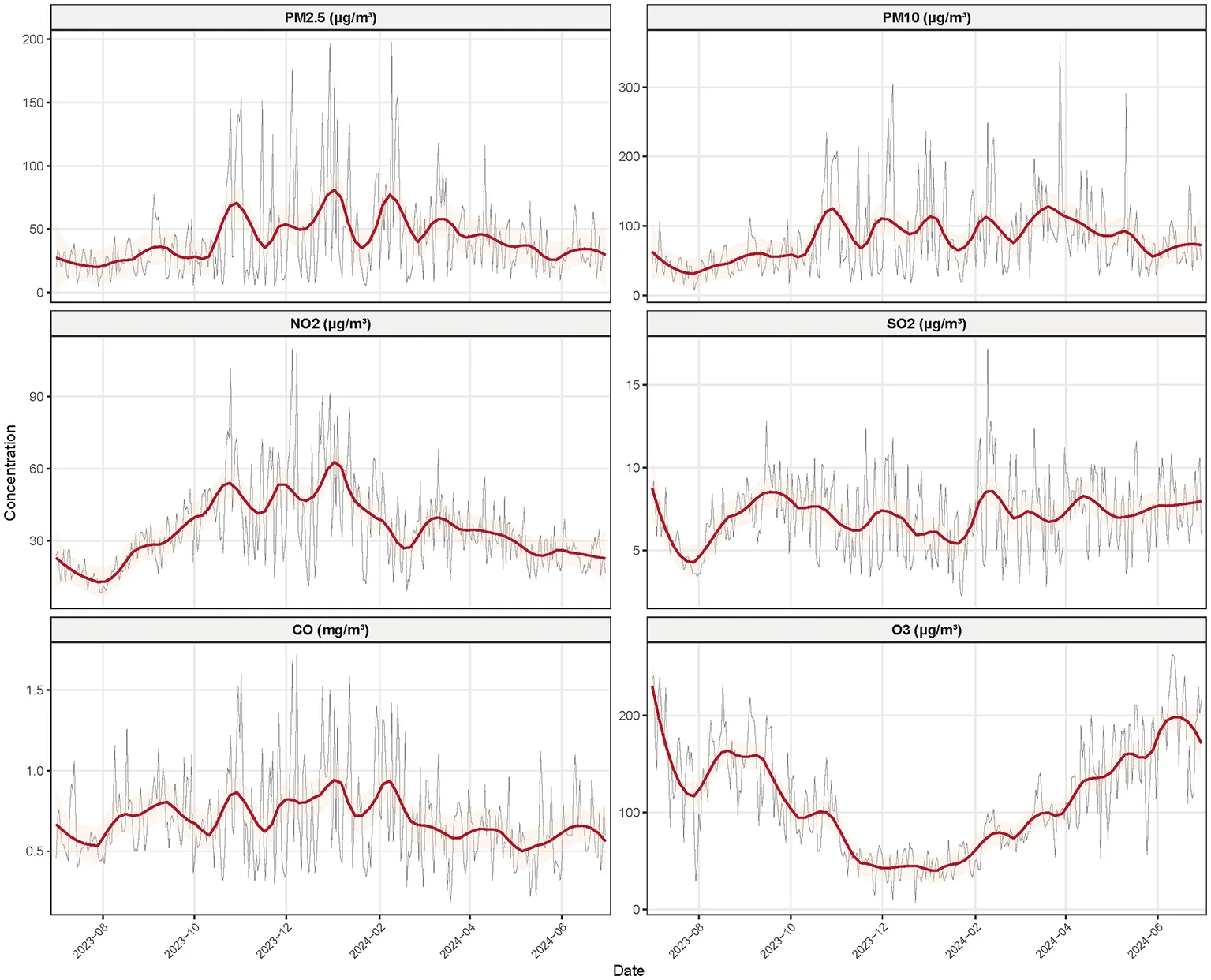
Daily concentrations of six major air pollutants with seasonal patterns. Gray lines represent daily concentrations of PM2.5, PM10, NO_2_, SO_2_, CO, and O3. Red LOESS-smoothed curves highlight seasonal patterns, with PM_2_.5, PM10, NO_2_, and CO peaking in winter, while O3 peaks in summer.

Air pollution levels also demonstrated strong seasonal variation. PM2.5, PM10, NO_2_, and CO concentrations were substantially higher in winter, with PM2.5 reaching a maximum of 197.6 μg/m^3^, whereas O3 displayed the opposite pattern, peaking during the summer months in accordance with photochemical formation processes (Figure 2). Descriptive statistics for all environmental variables are summarized in Table 1. Meteorological conditions varied widely, with daily mean temperatures ranging from −10.81 to 32.38 °C (mean 14.77 °C), reflecting the region's distinct four-season climate.

**Table 1:** Descriptive statistics of daily OHCA counts, air pollutants, and meteorological variables during the study period.

Variable	Mean	SD	Median	P25	P75	IQR	Min	Max
Daily OHCA count	21.9	6.1	21.1	17.3	26.0	8.8	7.0	42.1
PM2.5 (μg/m^3^)	42.2	33.8	33.7	19.8	51.9	32.1	4.6	197.6
PM10 (μg/m^3^)	80.2	53.5	66.4	42.7	98.0	55.1	7.4	364.6
NO_2_ (μg/m^3^)	34.8	17.9	31.1	22.2	43.7	21.7	8.4	110.3
SO_2_ (μg/m^3^)	7.0	2.2	7.1	5.2	8.6	3.4	2.2	17.2
CO (mg/m^3^)	0.7	0.3	0.6	0.5	0.8	0.3	0.2	1.7
O_3_ (μg/m^3^)	111.4	57.3	101.7	62.9	152.9	90.1	7.1	262.4
Mean temperature (°C)	14.8	11.8	17.0	3.3	25.3	22.4	−10.8	32.4
Min temperature (°C)	9.6	11.8	11.1	−1.9	20.4	22.4	−16.4	28.6
Max temperature (°C)	20.0	12.1	22.9	8.9	30.7	21.8	−7.7	40.2
Diurnal temperature range (°C)	10.4	3.6	10.4	7.9	12.8	4.9	1.9	18.6
Relative humidity (%)	56.8	17.4	56.8	43.1	70.6	27.5	14.1	93.7
Wind speed (m/s)	2.1	0.8	2.1	1.5	2.6	1.1	0.8	4.8
Atmospheric pressure (hPa)	1016.7	11.0	1015.7	1009.0	1025.0	16.0	991.2	1044.2

Correlation analyses revealed strong positive correlations among PM2.5, PM10, and CO, moderate correlations between NO_2_ and these pollutants, and negative correlations between O3 and most other pollutants, particularly NO_2_ (Spearman *r* = −0.505), consistent with known atmospheric chemistry. Full correlation matrices are provided in Supplementary Table S1 (Spearman) and Supplementary Table S2 (Pearson).

### Non-linear and lagged effects of temperature on OHCA risk

**Figure 3:**
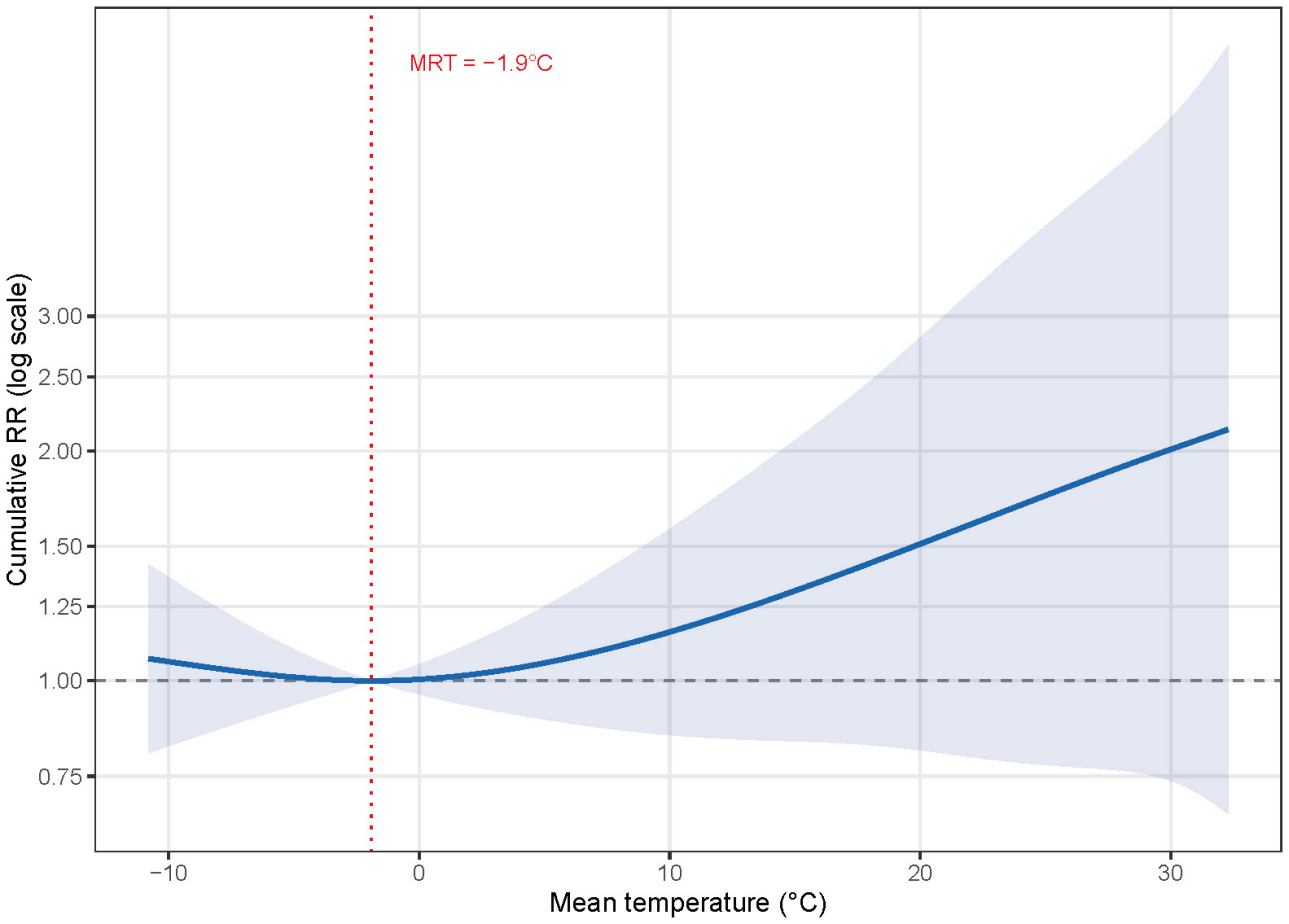
Overall Cumulative exposure–response curve for temperature and OHCA over a 14-Day lag window. Relative risks are expressed relative to the minimum risk temperature (−1.9 °C). The curve shows a monotonic increase in OHCA risk with rising temperatures, with wider confidence intervals at extreme heat.

The distributed lag non-linear model (DLNM) revealed a clear and biologically plausible pattern linking ambient temperature to OHCA risk. The minimum risk temperature (MRT) was identified at −1.9 °C, corresponding to the 10th percentile of the temperature distribution. Relative to this reference point, the cumulative exposure–response curve demonstrated a monotonic increase in OHCA risk across the temperature spectrum, with relative risks greater than 1 at higher temperatures but wide confidence intervals that include unity, indicating that the findings suggest a possible positive association rather than statistically significant evidence (Figure 3). Cumulative relative risks (RRs) at key temperature percentiles are presented in Table 2.

**Table 2:** Cumulative temperature-related relative risks (RR) across key percentiles over a 0–14 day lag period, with model specifications summarized in the table footnote.

Percentile	Temperature (°C)	Cumulative RR	95% CI (lower)	95% CI (upper)
1%	−9.1	1.049	0.841	1.309
5%	−4.0	1.005	0.954	1.058
10%	−1.9	1.000	1.000	1.000
25%	3.3	1.032	0.910	1.169
50%	17.0	1.385	0.829	2.316
75%	25.3	1.762	0.775	4.009
90%	28.9	1.949	0.756	5.025
95%	30.3	2.024	0.734	5.579
99%	31.7	2.099	0.693	6.361

This table presents cumulative RRs and 95% confidence intervals for selected temperature percentiles, referenced to the minimum risk temperature (MRT). A clear monotonic increase in cumulative RR is observed across the temperature distribution, with relative risks greater than 1 at higher temperatures. However, all corresponding confidence intervals include unity, indicating that the findings suggest a possible positive association rather than statistically significant evidence. Model parameters: Study period = 2023-07-01 to 2024-06-30; Total days = 366; Total OHCA cases = 7,996; Maximum lag = 14 days; Exposure knots = −1.92, 17.02, 28.86 °C; Lag knots = 0.91, 2.27, 5.64 days; Degrees of freedom for time trend = 7 per year; Minimum risk temperature (MRT) = −1.91 °C; Overdispersion parameter = 1.118.

At the low-temperature end, cumulative RRs remained close to unity and were not statistically significant, with RR = 1.049 (95% CI: 0.841–1.309) at the 1st percentile (−9.07 °C) and RR = 1.005 (95% CI: 0.954–1.058) at the 5th percentile (−3.96 °C). In contrast, high temperatures were associated with progressively increasing risk: RR = 1.385 (95% CI: 0.829–2.316) at the median temperature (17.02 °C), RR = 1.762 (95% CI: 0.775–4.009) at the 75th percentile (25.26 °C), and RR = 2.099 (95% CI: 0.693–6.361) at the 99th percentile (31.73 °C). Although confidence intervals widened at extreme temperatures due to fewer high-temperature days, the overall trend suggested a possible elevation in OHCA risk with increasing heat, but statistical significance was not achieved in the overall test (Wald test *p* = 0.12).

**Figure 4:**
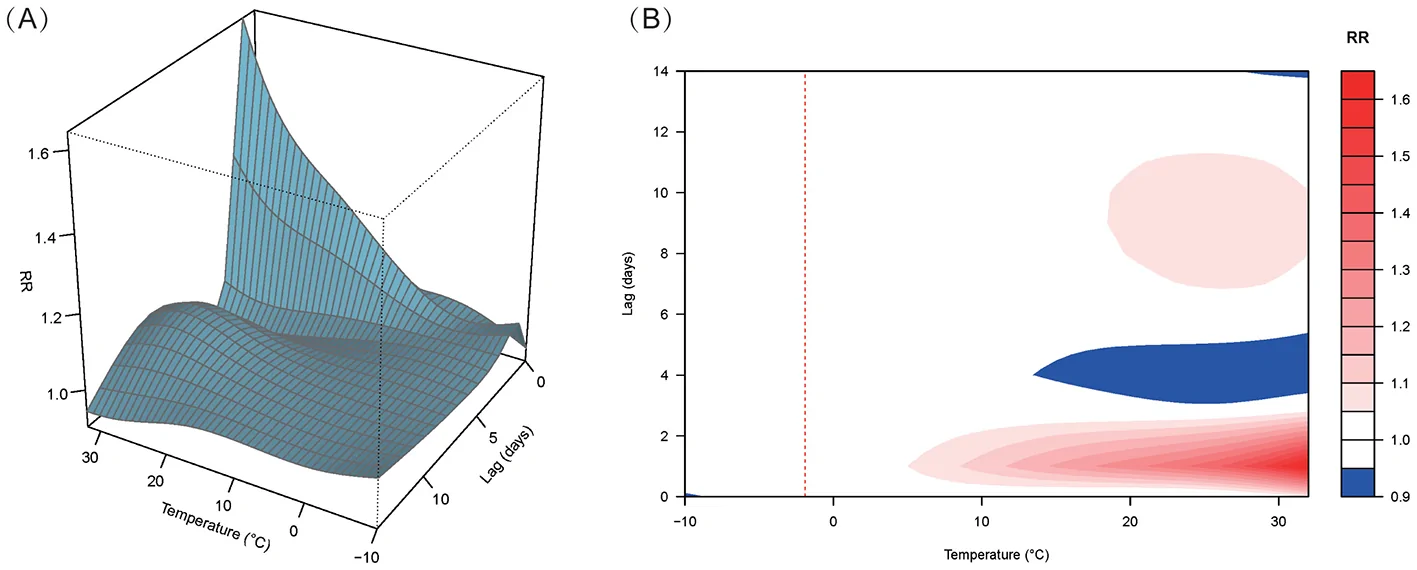
Three-dimensional exposure–lag–response surface for temperature and OHCA risk. **(A)** The 3D DLNM surface illustrates the joint effects of temperature and lag (0–14 days) on OHCA risk. High temperatures show strong short-term effects peaking at lag 1 day (RR > 1.5), while low temperatures show no meaningful association (RR ≈1.0). **(B)** Contour plot of temperature–lag–response relationship. Red regions indicate elevated relative risks (RR > 1.0), concentrated at high temperatures (>25 °C) and short lags (0–2 days). The dashed line marks the minimum risk temperature (−1.9 °C).

The three-dimensional exposure–lag–response surface further illustrated the acute nature of heat-related risk (Figure 4A). High temperatures produced a sharp increase in OHCA risk within the first 2 days following exposure, peaking at lag 1 day (RR > 1.5 at the 95th percentile temperature), followed by rapid attenuation to baseline by lag 3–4 days. A mild harvesting effect was observed at lags 4–5 days. In contrast, low temperatures produced no discernible lag pattern, with RR values remaining close to 1.0 across all 14 lag days. Lag-specific curves at extreme percentiles (Supplementary Figure S1) confirmed the absence of meaningful cold effects and the presence of a short, intense heat effect concentrated within 48 h.

The corresponding contour plot (Figure 4B) provided a complementary two-dimensional visualization. A distinct high-risk zone emerged at temperatures above approximately 25 °C, where a concentrated red band (RR > 1.0) appeared within the first 0–2 lag days, reinforcing the short-lived and intense nature of heat-related effects. Blue regions (RR < 1.0) dominated the low-temperature range, underscoring the absence of meaningful cold effects. The vertical dashed red line marking the MRT (−1.9 °C) aligned with the region where relative risks remained close to unity.

### Associations between air pollutants and OHCA risk

**Figure 5:**
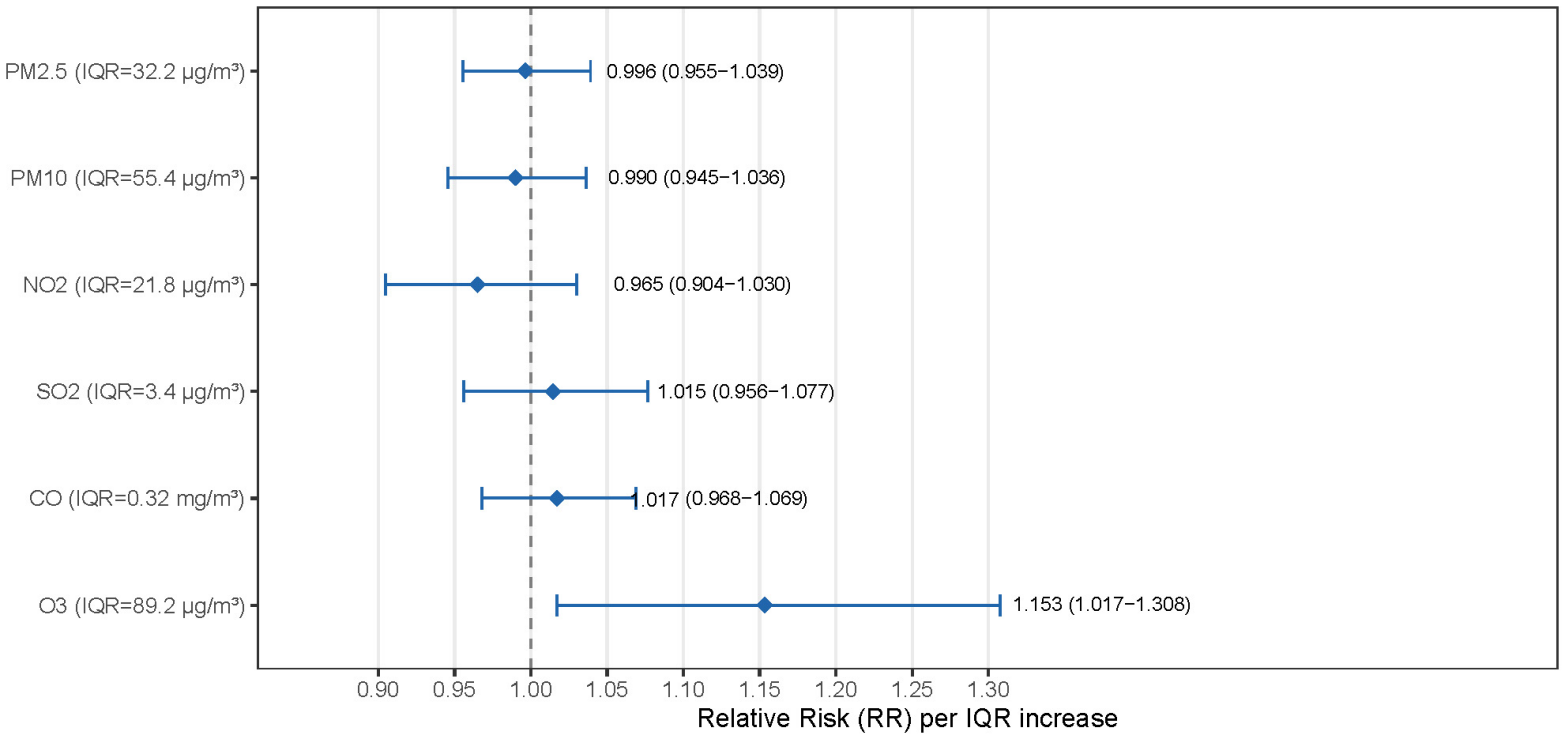
Forest plot of single-pollutant models. Relative risks (RR) and 95% confidence intervals for each pollutant per interquartile range (IQR) increase (lag0–1). Ozone is the only pollutant with a significant positive association with OHCA.

**Figure 6:**
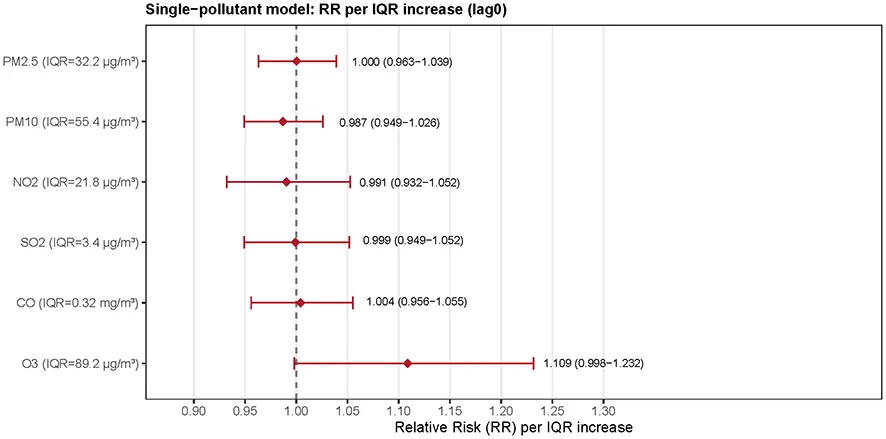
Forest plot of single-pollutant models (lag0). Relative risks (RR) and 95% confidence intervals for each pollutant per interquartile range (IQR) increase (lag0). Ozone (O3) shows the highest point estimate (RR = 1.109, 95% CI: 0.998–1.232), narrowly missing statistical significance, while all other pollutants (PM_2_.5, PM10, NO_2_, SO_2_, CO) exhibit null associations with OHCA risk.

In single-pollutant models adjusting for temperature (via DLNM), meteorological variables, long-term trends, day of week, and holidays, ozone (O3) emerged as the only pollutant significantly associated with OHCA risk. Each interquartile range (IQR) increase in lag0–1 O3 (89.2 μg/m^3^) corresponded to a 15.3% increase in OHCA incidence (RR = 1.153, 95% CI: 1.017–1.308, *p* = 0.027, Figure 5). The lag0 estimate for O3 showed a similar magnitude (RR = 1.109, 95% CI: 0.998–1.232, Figure 6) but narrowly missed statistical significance, suggesting that the 0–1 day exposure window better captures the short-term cardiovascular impact of ozone. Full single-pollutant results for both lag0 and lag0–1 windows are presented in Table 3.

**Table 3:** Single-pollutant model estimates for the associations between air pollutants and out-of-hospital cardiac arrest (OHCA) across lag0 and lag0–1 exposure windows.

Pollutant	Lag	IQR	RR	RR_low	RR_high	*P*_value
PM2.5	lag0-1	32.2	0.996	0.955	1.039	0.863
PM10	lag0-1	55.4	0.990	0.945	1.036	0.665
NO_2_	lag0-1	21.8	0.965	0.905	1.030	0.285
SO_2_	lag0-1	3.4	1.015	0.956	1.077	0.634
CO	lag0-1	0.32	1.017	0.968	1.069	0.504
O_3_	lag0-1	89.2	1.153	1.017	1.308	0.027
PM2.5	lag0	32.2	1.0004	0.963	1.0390	0.984
PM10	lag0	55.4	0.987	0.949	1.026	0.504
NO_2_	lag0	21.8	0.991	0.932	1.053	0.758
SO_2_	lag0	3.4	0.999	0.949	1.052	0.975
CO	lag0	0.32	1.004	0.956	1.055	0.864
O_3_	lag0	89.2	1.109	0.998	1.232	0.055

Other pollutants showed no significant associations across either lag structure. Forest plots (Figure 5) clearly illustrated that O3 was the only pollutant with a point estimate and confidence interval consistently above unity. Additional lag-window sensitivity analyses for all pollutants are provided in Supplementary Table S3.

### Two-pollutant models and independence of ozone effects

To evaluate whether the observed ozone effect was confounded by co-pollutants, we constructed three two-pollutant models including combinations of PM_2_.5, NO_2_, and O3. In all models, the ozone effect remained stable and statistically significant: RR = 1.161 (95% CI: 1.022–1.320) when adjusting for PM_2_.5, and RR = 1.148 (95% CI: 1.012–1.303) when adjusting for NO_2_. In contrast, PM_2_.5 and NO_2_ remained non-significant in all two-pollutant models. These findings, visualized in Supplementary Figure S2, indicate that the ozone–OHCA association is independent of particulate matter and nitrogen dioxide, consistent with the relatively weak correlations between O3 and other pollutants (Supplementary Tables S3, S4).

### Sensitivity analyses

Sensitivity analyses demonstrated strong robustness of the primary findings. When varying the maximum lag for temperature, the cumulative RR at the 95th percentile temperature remained elevated across lag windows of 7, 14, and 21 days (RR = 1.752, 2.024, and 1.442, respectively). Adjusting the degrees of freedom for long-term trends produced similarly stable results, with high-temperature cumulative RRs ranging from 1.681 to 2.024. Excluding extreme temperature days yielded a cumulative RR of 1.576 (95% CI: 0.559–4.444). These temperature-related sensitivity analyses are summarized in Supplementary Table S4.

For pollutants, ozone demonstrated a clear dose–lag pattern, with increasing RRs across lag0 (1.111), lag1 (1.101), lag0–1 (1.156), and lag0–3 (1.224), with the latter two reaching statistical significance. Other pollutants remained null across all lag structures (Supplementary Table S2). Collectively, these analyses confirm that the observed heat and ozone effects are not artifacts of modeling choices or extreme values.

### Seasonal stratification of temperature effects

Seasonal analyses revealed distinct temperature–OHCA associations in cold and warm seasons. In the cold season (November–March), the MRT was 3.89 °C, and the cumulative exposure–response curve showed a gradual increase in risk at both low and moderately high temperatures, although confidence intervals were wide due to reduced sample size. In the warm season (May–September), the MRT shifted to 23.47 °C, and the curve exhibited a pronounced upward inflection at high temperatures (≥30 °C), consistent with the acute heat effects observed in the main analysis. These seasonal differences are illustrated in Supplementary Figure S3, suggesting that heat-related OHCA risk is particularly pronounced during warm months, whereas cold-season effects are weaker and less consistent.

### Age- and sex-stratified analyses

**Figure 7:**
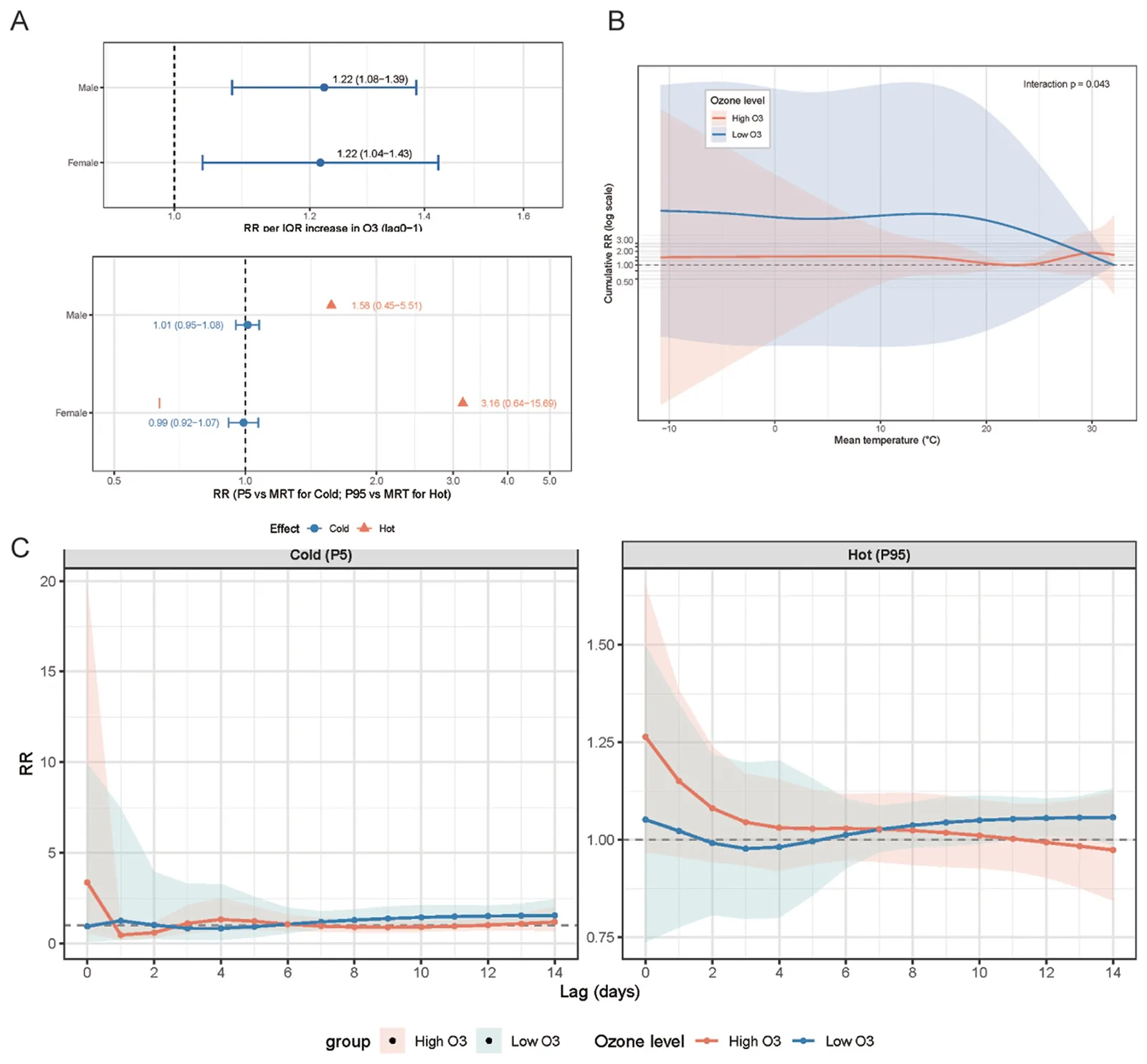
Stratified and interaction analyses of temperature and ozone in relation to OHCA. **(A)** Sex-stratified relative risks (RRs) for cold (P5 vs. MRT, blue circles), heat (P95 vs MRT, red triangles), and ozone exposure (per IQR increase in lag0–1 O3). **(B)** Temperature–ozone interaction curves. At the 95th percentile temperature, cumulative risk was higher in the high-ozone stratum (RR = 1.85, 95% CI: 0.59–5.79) than in the low-ozone stratum (RR = 1.49, 95% CI: 0.57–3.94); interaction testing confirmed significance (*p* = 0.043). **(C)** Lag-response functions at extreme temperatures. Under hot conditions, the high-ozone group showed a short-term peak at lag 1 day (RR = 1.15, 95% CI: 0.96–1.38), whereas the low-ozone group showed no significant change (RR = 1.02, 95% CI: 0.78–1.35). Under cold conditions, both strata yielded unstable estimates with wide confidence intervals (low ozone: RR = 13.00, 95% CI: 0.02–9081.84; high ozone: RR = 1.52, 95% CI: 0.01–306.16), indicating no reliable evidence of interaction.

In sex-specific analyses, ozone exposure showed consistent and significant associations in both men and women. Each IQR increase in lag0–1 O3 corresponded to a 22% rise in OHCA incidence (RR = 1.22, 95% CI: 1.08–1.39 in men; RR = 1.22, 95% CI: 1.04–1.43 in women), indicating comparable effect sizes across sexes (Figure 7A upper panel; Table 4).

**Table 4:** Stratified and interaction analyses: cumulative RRs (95% CIs) by sex, age group, and ozone level.

Factor	Subgroup	Exposure	RR (95% CI)
Sex	Men	Ozone (per IQR increase)	1.22 (1.08–1.39)
	Women	Ozone (per IQR increase)	1.22 (1.04–1.43)
	Men	Cold (P5 vs. MRT)	1.01 (0.95–1.08)
	Women	Cold (P5 vs. MRT)	0.99 (0.92–1.07)
	Men	Heat (P95 vs. MRT)	1.58 (0.45–5.51)
	Women	Heat (P95 vs. MRT)	3.16 (0.64–15.69)
Age	≥75 years	Ozone (per IQR increase)	1.05 (0.25–4.37)
	< 65 years	Ozone (per IQR increase)	Not converged (RR unstable)
	65–74 years	Ozone (per IQR increase)	Not converged (RR unstable)
Ozone–temperature interaction	Low ozone group	Cold (P5 vs. MRT)	13.00 (0.02–9081.84)
	High ozone group	Cold (P5 vs. MRT)	1.52 (0.01–306.16)
	Low ozone group	Heat (P95 vs. MRT)	1.49 (0.57–3.94)
	High ozone group	Heat (P95 vs. MRT)	1.85 (0.59–5.79)
	Low ozone group	Lag effect (Heat, lag1)	1.02 (0.78–1.35)
	High ozone group	Lag effect (Heat, lag1)	1.15 (0.96–1.38)
Interaction *p*-value	Temperature × Ozone	Continuous cross-product term	*p* = 0.043

RR, Relative Risk; CI, Confidence Interval; IQR, Interquartile Range; MRT, Minimum Risk Temperature; P5, 5th Percentile of Temperature Distribution; P95, 95th Percentile of Temperature Distribution; lag1, One-Day Lag.

By contrast, temperature effects differed by sex. Cold exposure (P5 vs. MRT) did not show meaningful associations (men: RR = 1.01; women: RR = 0.99). High temperatures (P95 vs. MRT) suggested a stronger risk elevation in women (RR = 3.16, 95% CI: 0.64–15.69) compared with men (RR = 1.58, 95% CI: 0.45–5.51), although wide confidence intervals limited statistical certainty (Figure 7A lower panel; Table 4).

In age-stratified analyses, model convergence was problematic in the < 65 and 65–74-year groups due to sparse daily OHCA counts. The distributed lag non-linear models failed to reach convergence standards, yielding unstable or infinite RR estimates and implausible confidence intervals. Consequently, results for these subgroups were not statistically reliable and are presented only as exploratory findings without inferential interpretation. In the ≥75-year group, the model converged, and ozone exposure per IQR increase showed a positive risk trend (RR = 1.05, 95% CI: 0.25–4.37). However, the wide confidence interval reflects limited precision, consistent with small sample sizes (Table 4). Similarly, age-stratified temperature curves did not converge, with RR estimates tending toward infinity (data not shown).

### Temperature–ozone interaction

At moderate temperatures (< 30 °C), risks were slightly higher in the low-ozone group, but wide confidence intervals suggested statistical uncertainty. High temperatures exerted stronger effects under elevated ozone conditions. At the 95th percentile temperature, cumulative risk was higher in the high-ozone stratum (RR = 1.85, 95% CI: 0.59–5.79) than in the low-ozone stratum (RR = 1.49, 95% CI: 0.57–3.94) (Figure 7B, Table 4).

Interaction analyses demonstrated that ozone amplified the cardiovascular impact of heat, with a significant interaction (*p* = 0.043). Stratum-specific estimates were imprecise, and the *p*-value was not adjusted for multiple testing; therefore, these results should be interpreted cautiously as exploratory evidence. Lag-response analyses further suggested a possible short-term synergy: under hot conditions, the high-ozone group showed a short-term peak at lag 1 day (RR = 1.15, 95% CI: 0.96–1.38), whereas the low-ozone group showed no significant change (RR = 1.02, 95% CI: 0.78–1.35) (Figure 7C; Table 4). Under cold conditions, ozone–temperature interaction models produced unstable estimates with extremely wide confidence intervals (low ozone: RR = 13.00, 95% CI: 0.02–9081.84; high ozone: RR = 1.52, 95% CI: 0.01–306.16), indicating no statistically reliable evidence of interaction.

## Discussion

In this population-based time-series study conducted in Tianjin, China, we found that short-term environmental exposures—particularly elevated ozone concentrations and possibly high ambient temperatures—were significantly associated with increased risk of OHCA. Using a distributed lag non-linear model, we demonstrated that heat exerted an acute and transient effect on OHCA incidence, peaking within 1, 2 days after exposure. In contrast, low temperatures showed no meaningful association across a 14-day lag window. Among six major air pollutants, ozone was the only pollutant consistently associated with OHCA risk, and its effect remained robust across multiple lag structures, sensitivity analyses, and two-pollutant models. These findings highlight the importance of heat and ozone as short-term cardiovascular triggers in northern Chinese cities with substantial seasonal variability.

The observed possible heat-related increase in OHCA risk aligns with prior studies reporting that high temperatures can precipitate acute cardiovascular events through dehydration, increased blood viscosity, electrolyte imbalance, thermoregulatory stress, and autonomic dysfunction (17, 18). The sharp peak at lag 1 day suggests rapid physiological decompensation, potentially via arrhythmogenic pathways or increased myocardial oxygen demand. The attenuationafter lag 2 days is consistent with the acute nature of heat stress and mirrors findings from Japan, Australia, and China (19–21). Nevertheless, wide confidence intervals crossing unity indicate limited statistical certainty. These findings should be interpreted as exploratory evidence rather than definitive proof that heat acutely triggers OHCA.

The absence of a significant cold effect contrasts with studies from colder regions, such as South Korea, where prolonged low temperatures substantially increase OHCA risk (22). Regional climatic and infrastructural differences likely explain this discrepancy: Tianjin's relatively milder winters, widespread indoor heating, and behavioral adaptation may buffer cold exposure. Moreover, the pronounced winter peak in OHCA observed in our datasetis more plausibly attributable to concurrent influenza epidemics, respiratory infections, and seasonal lifestyle factors that independently elevate cardiovascular risk, rather than to ambient cold itself (23, 24).

Stratified analyses provided further insight into susceptible populations, but several limitations must be acknowledged. In sex-specific analyses, ozone effects were consistent and significant in both men and women, with each IQR increase in lag0–1 O3 corresponding to a 22% rise in OHCA incidence. In contrast, temperature effects were less certain, with wide confidence intervals at extreme percentiles. High temperatures suggested a stronger risk elevation in women (RR = 3.16) compared with men (RR = 1.58), but the precision of these estimates was limited.

Age-specific analyses were more problematic. Models for the < 65 and 65–74-year groups failed to converge due to sparse daily OHCA counts, yielding unstable or implausible RR estimates. Consequently, no reliable conclusions could be drawn for these subgroups. In the ≥75-year group, the model converged and ozone exposure per IQR increase showed a positive risk trend (RR = 1.05), but the wide confidence interval (0.25–4.37) reflected low precision. These limitations underscore the need for larger datasets to validate age-specific effects (Table 4).

Interaction analyses further suggested that ozone may amplify the cardiovascular impact of heat. At the 95th percentile temperature, cumulative risk was higher in the high-ozone stratum (RR = 1.85) than in the low-ozone stratum (RR = 1.49), and formal interaction testing yielded *p* = 0.043. However, stratum-specific estimates were imprecise, and the analysis was not corrected for multiple comparisons. Therefore, this finding should be interpreted as exploratory evidence rather than conclusive proof of synergy.

Lag-response analyses showed that this synergy was concentrated in the immediate lag period: under hot conditions, the high-ozone group exhibited a short-term peak at lag 1 day (RR = 1.15), whereas the low-ozone group showed no significant change (RR = 1.02). These results suggest that ozone and heat may jointly destabilize cardiovascular physiology, consistent with mechanistic evidence linking oxidative stress and thermoregulatory strain to arrhythmogenesis.

The strong and independent association between ozone and OHCA is notable. Ozone, a secondary pollutant formed through photochemical reactions, peaks in summer when temperatures and solar radiation are highest. Biological mechanisms may involve oxidative stress, systemic inflammation, endothelial dysfunction, and autonomic imbalance, all of which can increase susceptibility to arrhythmias and sudden cardiac arrest (25–27). Our findings echo prior evidence from the United States, Europe and China, implicating ozone in arrhythmias, emergency cardiovascular admissions, and sudden cardiac death (28–30). The robustness of ozone effects across lag structures and two-pollutant models underscores its independence from particulate matter and nitrogen dioxide.

Given that ozone was the only pollutant significantly associated with OHCA, we further explored the potential interaction between temperature and ozone. Stratified analyses suggested that high temperatures may exert stronger effects under elevated ozone concentrations, consistent with biological plausibility that oxidative stress and thermoregulatory strain may jointly destabilize cardiovascular function (31, 32). Although our study period was limited to 1 year, these findings highlight the need for future multi-year investigations to quantify the combined impacts of heat and ozone on OHCA risk.

Ozone's unique photochemical behavior and distinct exposure pattern may make it a more potent short-term trigger compared with primary pollutants.

We did not observe significant associations between OHCA and PM_2_.5, PM10, NO_2_, SO_2_, or CO. Null or inconsistent results for particulate matter have been reported elsewhere (14, 33–36). Possible explanations include the relatively short study period, strong seasonal OHCA patterns overshadowing weaker pollution signals, and reduced residual confounding due to DLNM adjustment. Ozone's unique photochemical behavior and distinct exposure pattern may make it a more potent short-term trigger compared with primary pollutants.

Sensitivity analyses reinforce the robustness of our findings. Heat effects persisted across alternative lag windows, and exclusion of extreme days. Ozone effect demonstrated a clear dose–lag pattern and remained stable in two-pollutant models. Seasonal analyses revealed that heat-related OHCA risk was concentrated in the warm season, whereas cold-season effects were weaker and less consistent.

This study has several strengths, including comprehensive EMS coverage, DLNM modeling and extensive sensitivity analyses.

Limitations include potential exposure misclassification, the 1-year study period, and lack individual-level risk factor data The limited duration of 366 daily observations reduces statistical power and increases the risk of model overfitting, particularly in subgroup and interaction analyses. We therefore interpret these exploratory analyses with caution and emphasize the need for multi-year datasets to validate our findings.

## Conclusion

Elevated ozone concentrations were identified as important short-term environmental triggers of OHCA in Tianjin. High ambient temperatures showed a possible short-term association with limited statistical certainty. These findings highlight the need for targeted public health interventions during heatwaves and high-ozone episodes, including early warning systems, public education, and enhanced EMS preparedness. Future research should extend analyses over longer time periods, explore potential effect modifiers, and investigate the combined impacts of temperature, humidity, and air pollution.

## Data Availability

The raw data supporting the conclusions of this article will be made available by the authors, without undue reservation.
