# Supplementary material for: Ozone and possible heat effects on out-of-hospital cardiac arrest: a time-series study
Source: Front Public Health. 2026 Aug 20;14:1871041. doi: 10.3389/fpubh.2026.1871041 (PMC13538861; doi:10.3389/fpubh.2026.1871041)
Supplement: Supplementary file 1 [file Data_Sheet_1.zip › supplementary material/supplementary Tables.docx]

Table S1. Spearman correlation coefficients among air pollutants.

| Pollutant | PM2.5 | PM10 | NO2 | SO2 | CO | O3 |
| --- | --- | --- | --- | --- | --- | --- |
| PM2.5 | 1 | 0.904 | 0.572 | 0.442 | 0.727 | -0.075 |
| PM10 | 0.904 | 1 | 0.578 | 0.504 | 0.572 | -0.057 |
| NO2 | 0.572 | 0.578 | 1 | 0.419 | 0.551 | -0.505 |
| SO2 | 0.442 | 0.504 | 0.419 | 1 | 0.533 | 0.209 |
| CO | 0.727 | 0.572 | 0.551 | 0.533 | 1 | -0.123 |
| O3 | -0.075 | -0.057 | -0.505 | 0.209 | -0.123 | 1 |

Note：This table reports Spearman rank correlations between the six major air pollutants. Strong positive correlations were observed among PM₂.₅, PM₁₀, and CO, while NO₂ showed moderate correlations with particulate matter and CO. Ozone (O₃) exhibited negative correlations with most pollutants—particularly NO₂—consistent with known atmospheric photochemical processes. These correlation patterns informed the selection and interpretation of two‑pollutant models.

Table S2. Pearson correlation coefficients among air pollutants.

| Pollutant | PM2.5 | PM10 | NO2 | SO2 | CO | O3 |
| --- | --- | --- | --- | --- | --- | --- |
| PM2.5 | 1 | 0.867 | 0.685 | 0.394 | 0.774 | -0.174 |
| PM10 | 0.867 | 1 | 0.607 | 0.39 | 0.583 | -0.121 |
| NO2 | 0.685 | 0.607 | 1 | 0.386 | 0.648 | -0.491 |
| SO2 | 0.394 | 0.39 | 0.386 | 1 | 0.497 | 0.208 |
| CO | 0.774 | 0.583 | 0.648 | 0.497 | 1 | -0.163 |
| O3 | -0.174 | -0.121 | -0.491 | 0.208 | -0.163 | 1 |

Note: This table presents Pearson correlation coefficients for the six pollutants, demonstrating patterns similar to those observed in the Spearman analysis. PM₂.₅, PM₁₀, and CO were strongly correlated, while O₃ remained negatively correlated with most primary pollutants. These findings further support the independence of ozone effects observed in the single‑ and two‑pollutant models.

Table S3. Sensitivity analyses of pollutant effects across alternative lag windows.

| Analysis | Pollutant | Setting | IQR | RR | RR_low | RR_high |
| --- | --- | --- | --- | --- | --- | --- |
| Lag window | PM2.5 | Lag 0 | 32.4 | 1.0004 | 0.963 | 1.0391 |
| Lag window | PM2.5 | Lag 1 | 32.4 | 0.9965 | 0.9684 | 1.0255 |
| Lag window | PM2.5 | Lag 0-1 | 32.4 | 0.9965 | 0.9552 | 1.0395 |
| Lag window | PM2.5 | Lag 0-3 | 32.4 | 1.0089 | 0.9542 | 1.0668 |
| Lag window | PM10 | Lag 0 | 55.4 | 0.9872 | 0.9494 | 1.0264 |
| Lag window | PM10 | Lag 1 | 55.4 | 0.9996 | 0.968 | 1.0324 |
| Lag window | PM10 | Lag 0-1 | 55.4 | 0.9907 | 0.9463 | 1.0372 |
| Lag window | PM10 | Lag 0-3 | 55.4 | 1.0016 | 0.9432 | 1.0637 |
| Lag window | NO2 | Lag 0 | 21.8 | 0.9894 | 0.931 | 1.0514 |
| Lag window | NO2 | Lag 1 | 21.8 | 0.9722 | 0.9302 | 1.016 |
| Lag window | NO2 | Lag 0-1 | 21.8 | 0.9639 | 0.9033 | 1.0286 |
| Lag window | NO2 | Lag 0-3 | 21.8 | 0.967 | 0.894 | 1.0459 |
| Lag window | SO2 | Lag 0 | 3.4 | 1.0002 | 0.9502 | 1.0528 |
| Lag window | SO2 | Lag 1 | 3.4 | 1.0148 | 0.9724 | 1.0591 |
| Lag window | SO2 | Lag 0-1 | 3.4 | 1.0145 | 0.9559 | 1.0767 |
| Lag window | SO2 | Lag 0-3 | 3.4 | 1.0289 | 0.9528 | 1.111 |
| Lag window | CO | Lag 0 | 0.32 | 1.0041 | 0.9557 | 1.055 |
| Lag window | CO | Lag 1 | 0.32 | 1.0125 | 0.9799 | 1.0461 |
| Lag window | CO | Lag 0-1 | 0.32 | 1.0166 | 0.9672 | 1.0686 |
| Lag window | CO | Lag 0-3 | 0.32 | 1.027 | 0.9675 | 1.0902 |
| Lag window | O3 | Lag 0 | 88.4 | 1.1108 | 1.001 | 1.2327 |
| Lag window | O3 | Lag 1 | 88.4 | 1.1008 | 0.9921 | 1.2213 |
| Lag window | O3 | Lag 0-1 | 88.4 | 1.1561 | 1.0205 | 1.3097 |
| Lag window | O3 | Lag 0-3 | 88.4 | 1.2236 | 1.0568 | 1.4166 |

Note: This table summarizes relative risks (RRs) and 95% confidence intervals for each interquartile range (IQR) increase in pollutant concentrations across multiple lag structures (lag0, lag1, lag0–1, and lag0–3). Ozone (O₃) consistently demonstrated the strongest and most coherent dose–lag pattern, with statistically significant associations observed for lag0–1 and lag0–3 windows. In contrast, PM₂.₅, PM₁₀, NO₂, SO₂, and CO showed no significant associations across any lag specification, reinforcing the conclusion that ozone is the only pollutant exerting a measurable short‑term impact on OHCA risk.

Table S4. Sensitivity analyses for temperature model specifications.

| Analysis | Pollutant | Setting | IQR | RR | RR_low | RR_high | Analysis |
| --- | --- | --- | --- | --- | --- | --- | --- |
| Lag window | PM2.5 | Lag 0 | 32.4 | 1.0004 | 0.963 | 1.0391 | Lag window |
| Lag window | PM2.5 | Lag 1 | 32.4 | 0.9965 | 0.9684 | 1.0255 | Lag window |
| Lag window | PM2.5 | Lag 0-1 | 32.4 | 0.9965 | 0.9552 | 1.0395 | Lag window |
| Lag window | PM2.5 | Lag 0-3 | 32.4 | 1.0089 | 0.9542 | 1.0668 | Lag window |
| Lag window | PM10 | Lag 0 | 55.4 | 0.9872 | 0.9494 | 1.0264 | Lag window |
| Lag window | PM10 | Lag 1 | 55.4 | 0.9996 | 0.968 | 1.0324 | Lag window |
| Lag window | PM10 | Lag 0-1 | 55.4 | 0.9907 | 0.9463 | 1.0372 | Lag window |
| Lag window | PM10 | Lag 0-3 | 55.4 | 1.0016 | 0.9432 | 1.0637 | Lag window |

Note: This table presents the results of sensitivity analyses evaluating the robustness of temperature–OHCA associations under alternative model specifications. Different lag windows (lag0, lag1, lag0–1, and lag0–3) were applied to assess whether the estimated effects of temperature remained stable across varying temporal assumptions. Across all settings, effect estimates remained consistent in direction and magnitude, supporting the robustness of the primary DLNM temperature findings.
